# Comparison of the Effectiveness of Various Medicines in the Prevention of Ovarian Hyperstimulation Syndrome: A Network Meta-Analysis of Randomized Controlled Trials

**DOI:** 10.3389/fendo.2022.808517

**Published:** 2022-01-26

**Authors:** Di Wu, Hao Shi, Yiping Yu, Ting Yu, Jun Zhai

**Affiliations:** ^1^ Center for Reproductive Medicine, The First Affiliated Hospital of Zhengzhou University, Zhengzhou, China; ^2^ Henan Key Laboratory of Reproduction and Genetics, The First Affiliated Hospital of Zhengzhou University, Zhengzhou, China; ^3^ Henan Provincial Obstetrical and Gynecological Diseases (Reproductive Medicine) Clinical Research Center, The First Affiliated Hospital of Zhengzhou University, Zhengzhou, China

**Keywords:** ovarian hyperstimulation syndrome, various drugs, controlled ovulation stimulation, network meta-analysis, OHSS

## Abstract

**Background:**

Previous studies have described the effects of different drugs in preventing ovarian hyperstimulation syndrome (OHSS). However, the efficacies of those drugs in preventing OHSS remain inconclusive.

**Methods:**

We searched the PubMed, Web of Science, Embase, and Cochrane Central Register of Controlled Trials (CENTRAL) databases. A network meta-analysis of randomized controlled trials (RCTs) was performed up to August 2021. We investigated the following drugs in our study: aspirin, albumin, metformin, calcium, cabergoline, quinagolide, letrozole, hydroxyethyl starch (HES), and glucocorticoids. The primary outcome was the incidence rate of moderate-to-severe OHSS, with the results presented as risk ratios (RRs) with 95% confidence intervals (CIs).

**Results:**

The incidence of moderate-to-severe OHSS was significantly reduced by calcium administration (risk ratios [RR] 0.14, 95% confidence interval [CI]: 0.04, 0.46) (grade: high), HES (RR 0.25, 95% CI 0.07, 0.73) (grade: high), and cabergoline (RR 0.43, 95% CI 0.24, 0.71) (grade: moderate). The surface under the cumulative ranking curve (SUCRA) indicated that calcium (SUCRA, 92.4%) was the most effective intervention for preventing moderate-to-severe OHSS. These drugs were safe and did not affect clinical pregnancy, miscarriage, or live birth rates.

**Conclusion:**

Calcium, HES, and cabergoline could effectively and safely prevent moderate-to-severe OHSS, with calcium as the most effective intervention.

## Introduction

Ovarian hyperstimulation syndrome (OHSS) is a common complication of controlled ovulation stimulation (COS). A review reported that the incidence of OHSS was 6%–11% for *in vitro* fertilisation or intracytoplasmic sperm injection (IVF/ICSI) (1). To date, several risk factors have been reported for OHSS including high serum anti-Mullerian hormone levels (> 3.4 ng/mL), high peak estradiol (E_2_) (> 3500 pg/mL), multiple follicle development (> 25), and a high number of retrieved oocytes (> 24) (2). Women with polycystic ovarian syndrome (PCOS) are at higher risks of developing OHSS during COS. In addition, our previous studies have shown that the incidence of OHSS is related to temperature and is higher in extreme climates (summer and winter) (3). Mild OHSS is characterised by slightly enlarged ovaries. Moderate OHSS is characterised by abdominal distension, nausea, vomiting, and diarrhoea. Pleural fluid, ascites, abnormal kidney function, and coagulation abnormalities, which could be life threatening, can be observed in severe OHSS patients (4). Furthermore, OHSS increases the cycle cancellation rate and financial burden, which prolongs the treatment time. Therefore, OHSS prevention is a key clinical concern.

Currently, several approaches have been used to prevent OHSS, such as using gonadotropin-releasing hormone (GnRH) antagonist protocols for COS, replacing the human chorionic gonadotropin (HCG) with GnRH-agonist for trigger, and replacing the fresh embryo transfers to whole embryo cryopreservation (2). Previous studies have described the effects of numerous drugs in preventing OHSS, including calcium (5–9), glucocorticoids (10–12), hydroxyethyl starch (HES) (13, 14), albumin (13, 15–23), aspirin (24, 25), cabergoline (6–9, 12, 15, 22, 23, 26–32), letrozole (24, 33), metformin (34–43), and quinagolide (31, 44). However, the efficacies of these drugs in preventing OHSS remain controversial or inconclusive.

Given the lack of direct comparisons among studies, the optimal drugs for preventing OHSS remain unclear. In this network meta-analysis, we present direct and indirect evidence regarding multiple drug comparisons (45). Furthermore, we considered the efficacy and safety of different drugs in preventing OHSS during COS. The aim of this study was to investigate drugs that can prevent OHSS and to compare the efficacy of different interventions to provide clinical guidance for women at high-risk of OHSS.

## Methods

### Inclusion and Exclusion Criteria

Our study population was at high risk of OHSS based on any of the following conditions: ≥ 20 retrieved oocytes, E2 > 3000 pg/mL on HCG day, ≥18 follicles on HCG day, and polycystic ovary syndrome (PCOS) or polycystic ovary (9, 18, 34, 38). We included the following drugs in our study: aspirin, albumin, metformin, calcium, cabergoline, quinagolide, letrozole, HES, glucocorticoids, bromocriptine, and progesterone. Placebo, blank group, or other drugs were used as controls. We excluded studies if treatment was performed using the same medicine, with only the dose and duration of use differing between groups. Moreover, we excluded studies that combined two or more drugs. The outcomes included the incidence rate of moderate-to-severe OHSS, clinical pregnancy rate, miscarriage rate, and live birth rate. We only included RCTs regarding the prevention of OHSS during IVF/ICSI, and excluded retrospective studies, reviews, case reports, conference papers, meta-analyses, duplicate published studies, studies lacking the required outcome indicators and incomplete data regarding outcome indicators, studies lacking the appropriate design, and studies with poor quality assessment. Since this study was a network meta-analysis, formal ethical approval was not required.

### Search Strategy and Screening

This study was performed according to the Preferred Reporting Items for Systematic Reviews and Meta-analyses (PRISMA) (46). We searched for eligible RCTs in the PubMed, EMBASE, Web of Science, and Cochrane Library databases published from database establishment to 22 August 2021. The search strategy and adjustment to the syntax for the databases are presented in **Supplemental 1**. Additionally, we searched for relevant articles in the references. Two researchers independently screened the articles (D. W. and H. S.), with disagreements being resolved by a third researcher (T. Y.).

### Data Extraction and Efficacy Measures

We designed a special table for this analysis after a thorough assessment of all studies. We extracted the following data: first author (year), country, demographic characteristics (inclusion criteria for patients, diagnostic criteria for OHSS, age, body mass index [BMI]), sample size, COS protocol, details regarding drug use, number of moderate-to-severe OHSS, number of clinical pregnancies, number of miscarriages, number of live births, risk bias, and randomisation method. Additionally, we contacted the authors *via* calls or emails to obtain original data.

### Primary and Secondary Outcomes

The primary outcome was the incidence rate of moderate-to-severe OHSS. Moderate-to-severe OHSS is mainly characterised by abdominal distension, nausea, vomiting, diarrhoea, hydrothorax, blood clotting disorders, and abnormal kidney function (4). Secondary outcomes included the clinical pregnancy rate, miscarriage rate, and live birth rate.

### Risk-of-Bias Assessment

The risk of bias was assessed based on the Cochrane Collaboration Handbook for Quality Assessment (47). The tools used to evaluate the risk of bias included the following factors: allocation concealment, random sequence generation, blinding of participants and personnel, blinding of outcome assessment, selective reporting, incomplete outcome data, and other sources of bias. Each iteration was assessed; additionally, the risk of bias was classified as low, high, or unclear. Since this was a meta-analysis, formal ethical approval was not required.

### Quality of Evidence

This meta-analysis assessed the quality of evidence-based on the Grading of Recommendations Assessment, Development, and Evaluation (GRADE) Working Group (48). The risk of bias, inconsistency (heterogeneity), imprecision, indirectness, and publication bias for pairwise comparisons were assessed. The quality of evidence was ranked as high (high confidence in the evidence), moderate (moderate confidence), low (some confidence), and very low (little confidence).

### Statistical Analysis

In this network-meta analysis, analyses were performed using the Bayesian theory. We used the following analytics and graphing software: R (version 4.1.1), Stata (version 14.0, Stata Corp LP, 4905 Lakeway Drive, College Station, TX, USA), and JAGS (version 4.3.0). Furthermore, we used the gemtc package (49). The network meta-analysis was performed *a priori* using a Markov chain Monte Carlo method. Moreover, we used a generalised linear model with four chains, performing 50,000 iterations followed by 10,000 adjustments. Inconsistency was assessed using the node split method, with the results being analysed and ranked using the consistency and non-consistency models if the difference was insignificant and significant (p > 0.05), respectively (50). The study outcomes were binary variables; furthermore, the results were expressed as risk ratios (RR) with 95% confidence intervals (CI). Statistical significance was set at P < 0.05. Among-study heterogeneity was assessed using the I^2^ test (0%–30%, mild heterogeneity; 30%–60%, moderate heterogeneity; > 60%, substantial heterogeneity). Fixed-effects and random-effects models were used when heterogeneity was absent and present, respectively. In the case of a closed loop, the heterogeneity results were expressed as RR and its 95% CI, with 1 (RR) and *P* > 0.05 representing no heterogeneity; otherwise, heterogeneity was present. Forest and league plots were used to present the results of pairwise comparisons in the network meta-analysis. To determine the ranking of drug efficacy, we used Bayesian analysis to obtain the surface under the cumulative ranking curve (SUCRA) (45, 51). The effectiveness of each intervention is presented as a percentage, which allows identification of the best intervention. The SUCRA value is directly proportional to the effectiveness of the intervention (51, 52). Publication bias was assessed using a comparison-adjusted funnel plot.

## Results

### Literature Search Results

**Figure 1:**
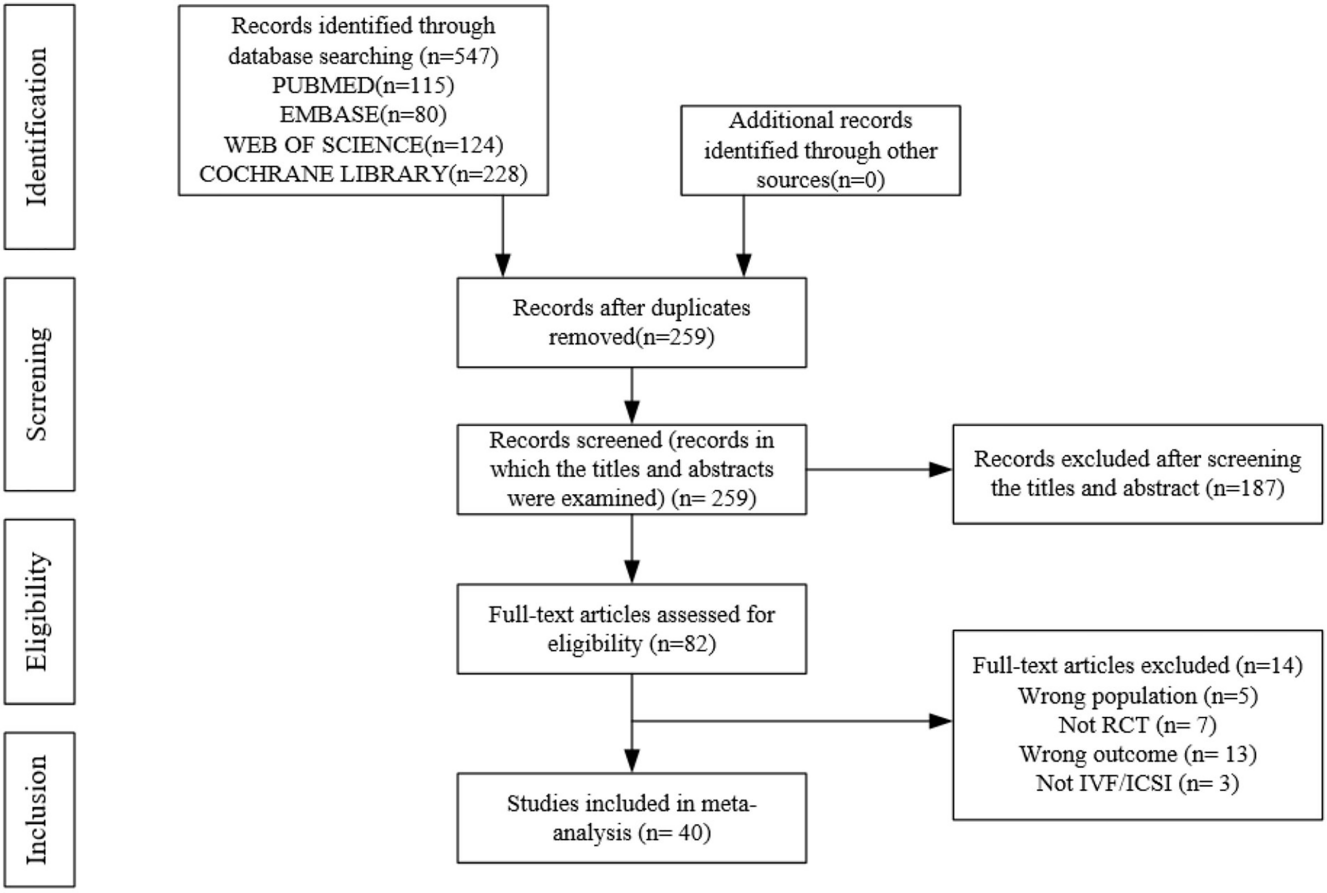
Flow chart.

A preliminary search of multiple databases yielded 259 articles. After browsing the titles and abstracts, 82 studies were included. After a full-text review, 40 articles (n = 5849) were included. **Figure 1** shows the flow chart. The included studies were presented in **Supplemental 2**.

### Characteristics of Included Studies

We did not analyse the effect of bromocriptine and progesterone on OHSS since they were not assessed in the included RCTs. Therefore, nine interventions were analysed, including aspirin, letrozole, cabergoline, albumin, HES, metformin, calcium, glucocorticoids, and quinagolide. Thirteen RCTs included women with PCOS. The included RCTs used various criteria for classifying OHSS (**Supplemental Table 1**); The definitions for moderate-to-severe OHSS were similar (**Supplemental 3.1**). Most RCTs used Golan’s criteria for OHSS classification (4). Only 31 studies (n = 4964) with clear OHSS classification criteria and distinct reporting of moderate-to-severe OHSS were included in the analysis of the preventative effects of drugs on moderate-to-severe OHSS. The remaining nine RCTs were only analysed for safety outcomes (7, 22, 35, 36, 38–41, 43). **Supplemental Table 1** presents the characteristics of the study population. The effects of eight drugs on clinical pregnancy were analysed in 29 RCTs (n = 3965). Cabergoline was the most frequently studied drug, followed by albumin and metformin. There were no significant differences of baseline characteristics (**Supplemental Table 1**).

### Quality of Evidence

**Table 1** shows the GRADE assessment of interventions for preventing moderate-to-severe OHSS. Nearly half of the evidence was moderate, with little evidence being low.

**Table 1:** Quality of evidence for moderate-to-severe OHSS.

Comparison	Indirect evidence	Direct evidence	Network meta-analysis	
	Certainty of evidence	Certainty of evidence	RR(95%CI)	Certainty of evidence
Aspirin: Placebo/blank	Moderate(risk of bias)	Moderate(risk of bias)	0.83 (0.21, 2.95)	Low(risk of bias, imprecision)
Aspirin: Letrozole	Moderate(risk of bias)	High	2.52 (0.7, 9.69)	High
Aspirin: Cabergoline	Low(risk of bias, publication bias)	NA	1.67 (0.38, 6.55)	Low(risk of bias, publication bias)
Aspirin: Albumin	Moderate(risk of bias)	NA	1.39 (0.32, 6.3)	Moderate(risk of bias)
Aspirin: HES	Moderate(risk of bias)	NA	3.22 (0.55, 18.87)	Moderate(risk of bias)
Aspirin: Metformin	Moderate(risk of bias)	NA	1.4 (0.27, 6.97)	Moderate(risk of bias)
Aspirin: Calcium	Moderate(risk of bias)	NA	5.14 (0.86, 30.1)	Moderate(risk of bias)
Aspirin: Glucocorticoids	Moderate(risk of bias)	NA	0.83 (0.14, 4.32)	Low(risk of bias, imprecision)
Aspirin: Quinagolide	Moderate(risk of bias)	NA	1.79 (0.28, 10.41)	Moderate(risk of bias)
Letrozole: Placebo	Moderate(publication bias)	Moderate(risk of bias)	0.33 (0.07, 1.26)	Moderate(publication bias)
Letrozole: Cabergoline	Moderate(publication bias)	NA	0.66 (0.13, 2.81)	Moderate(publication bias)
Letrozole: Albumin	High	High	0.55 (0.11, 2.62)	High
Letrozole: HES	High	High	1.28 (0.19, 8.08)	High
Letrozole: Metformin	Moderate(risk of bias)	NA	0.56 (0.09, 2.85)	Moderate(risk of bias)
Letrozole: Calcium	Moderate(risk of bias)	NA	2.03 (0.3, 12.97)	Moderate(risk of bias)
Letrozole: Glucocorticoids	Moderate(risk of bias)	NA	0.33 (0.05, 1.82)	Moderate(risk of bias)
Letrozole: Quinagolide	Moderate(risk of bias)	NA	0.7 (0.1, 4.27)	Moderate(risk of bias)
Cabergoline: Placebo	Moderate(risk of bias)	Low(risk of bias, publication bias)	0.5 (0.3, 0.83)	Moderate(risk of bias)
Cabergoline: Albumin	High	Moderate(publication bias)	0.84 (0.42, 1.92)	Moderate(imprecision)
Cabergoline: HES	Low(risk of bias, publication bias)	High	1.94 (0.61, 6.85)	High
Cabergoline: Metformin	Low(risk of bias, publication bias)	NA	0.85 (0.29, 2.59)	Very low(risk of bias, publication bias, imprecision)
Cabergoline: Calcium	Low(risk of bias, publication bias)	Moderate(risk of bias)	3.09 (1.01, 10.13)	Moderate(risk of bias)
Cabergoline: Glucocorticoids	Low(risk of bias, publication bias)	High	0.5 (0.16, 1.56)	High
Cabergoline: Quinagolide	Low(risk of bias, publication bias)	High	1.08 (0.32, 3.64)	Moderate(imprecision)
Albumin: Placebo	Low(risk of bias, publication bias)	Moderate(publication bias)	0.6 (0.28, 1.08)	Moderate(publication bias)
Albumin: HES	Moderate(publication bias)	High	2.32 (0.69, 7.63)	High
Albumin: Metformin	Moderate(publication bias)	NA	1.02 (0.31, 3.05)	Low(publication bias, imprecision)
Albumin: Calcium	Moderate(publication bias)	NA	3.69 (0.94, 13.67)	Moderate(publication bias)
Albumin: Glucocorticoids	Moderate(publication bias)	NA	0.6 (0.15, 1.97)	Moderate(publication bias)
Albumin: Quinagolide	Moderate(publication bias)	NA	1.29 (0.31, 4.75)	Moderate(publication bias)
HES: Placebo	Moderate(publication bias)	High	0.26 (0.08, 0.77)	High
HES: Metformin	High	NA	0.44 (0.09, 1.87)	High
HES: Calcium	Moderate(risk of bias)	NA	1.58 (0.31, 8.05)	Moderate(risk of bias)
HES: Glucocorticoids	High	NA	0.26 (0.05, 1.19)	High
HES: Quinagolide	High	NA	0.55 (0.1, 2.75)	High
Metformin: Placebo	NA	High	0.59 (0.22, 1.49)	High
Metformin: Calcium	High	NA	3.63 (0.79, 17.08)	High
Metformin: Glucocorticoids	High	NA	0.59 (0.14, 2.39)	High
Metformin: Quinagolide	High	NA	1.27 (0.26, 5.79)	High
Calcium: Placebo	Low(risk of bias, publication bias)	High	0.16 (0.05, 0.52)	High
Calcium: Glucocorticoids	Moderate(risk of bias)	NA	0.16 (0.03, 0.77)	Moderate(risk of bias)
Calcium: Quinagolide	Moderate(risk of bias)	NA	0.35 (0.06, 1.74)	Moderate(risk of bias)
Glucocorticoids: Placebo	Low(risk of bias, publication bias)	High	0.99 (0.35, 2.91)	Moderate(imprecision)
Glucocorticoids : Quinagolide	High	NA	2.15 (0.43, 10.88)	High
Quinagolide: Placebo	Low(risk of bias, publication bias)	High	0.46 (0.14, 1.56)	High

NA, Not Applicable.

### Primary Outcome Measure

**Figure 2:**
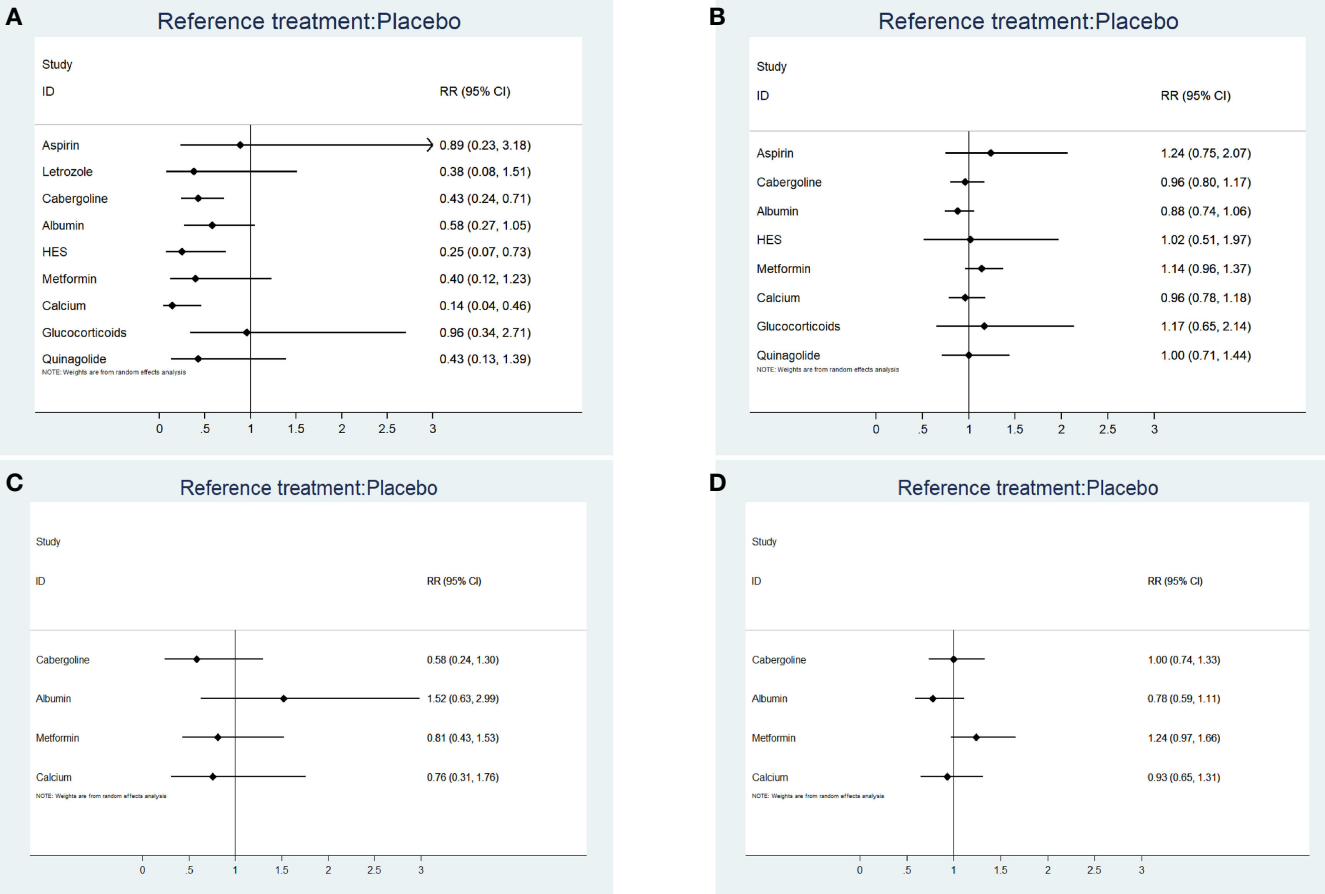
**(A)** The forest plot for incidence rate of moderate-to-severe OHSS. **(B)** The forest plot for clinical pregnancy rate. **(C)** The forest plot for miscarriage rate. **(D)** The forest plot for live birth rate.

**Supplemental 3.2** shows a network plot for preventing moderate-to-severe OHSS. Most studies on the preventative effects of drugs on moderate-to-severe OHSS directly compared interventions with blank/placebo groups. One study directly compared aspirin and letrozole (24), while two studies directly compared cabergoline and albumin (15, 22). Another study directly compared cabergoline and HES (22), while two studies directly compared cabergoline and calcium (6, 8). Additionally, one study directly compared cabergoline and glucocorticoids (12), while two studies directly compared HES and albumin (13, 22), and another study directly compared cabergoline and quinagolide (31). Calcium (RR 0.14, 95% CI 0.04, 0.46) (grade: high), HES (RR 0.25, 95% CI 0.07, 0.73) (grade: high), and cabergoline (RR 0.43, 95% CI 0.24, 0.71) (grade: moderate) significantly prevented moderate-to-severe OHSS compared with placebo or blank control. Contrastingly, letrozole (grade: moderate), aspirin (grade: low), albumin (grade: moderate), metformin (grade: high), glucocorticoids (grade: moderate), and quinagolide (grade: high) could not prevent moderate-to-severe OHSS (P > 0.05). **Figure 2A** shows the forest plot.

**Figure 3:**
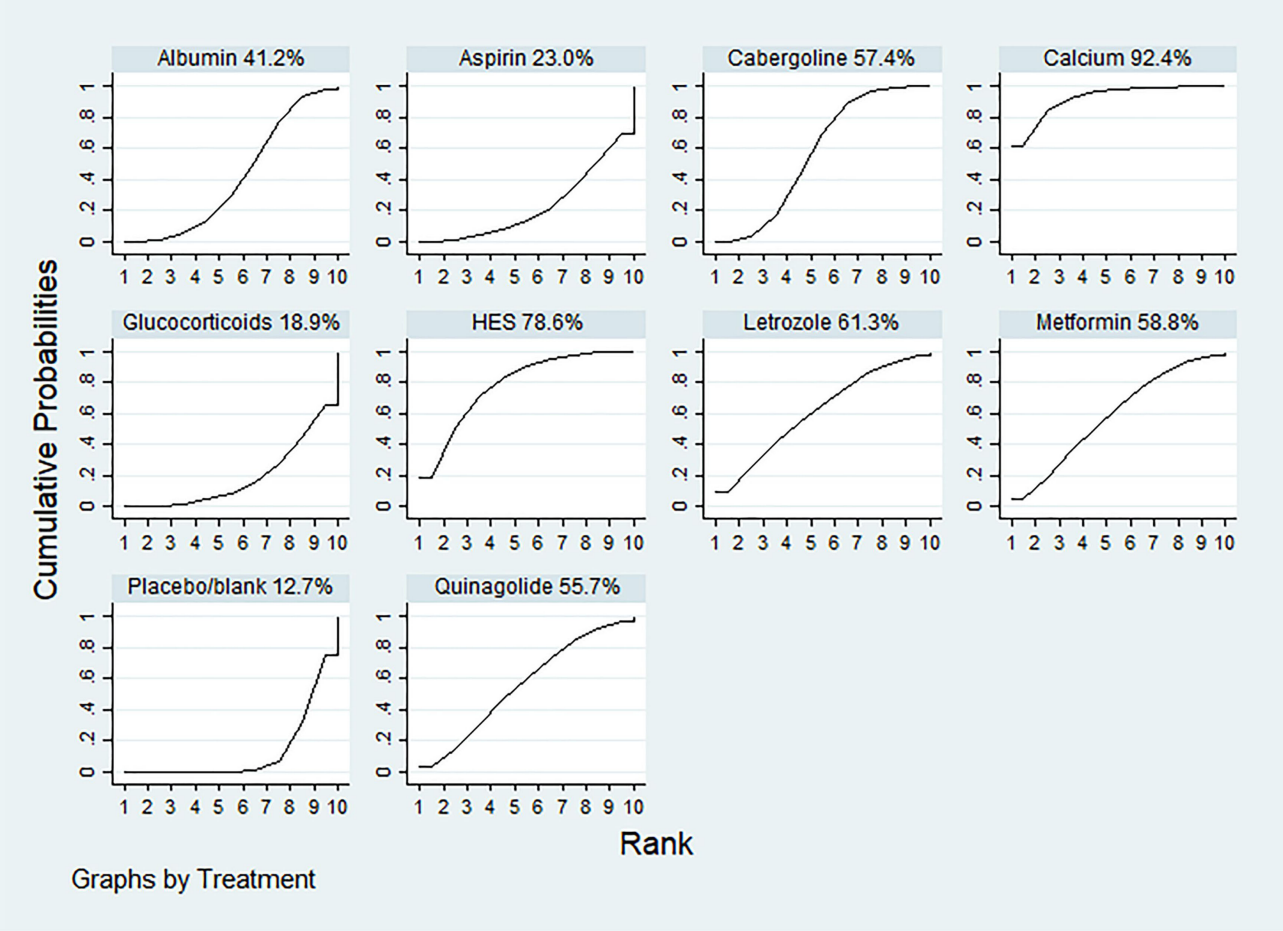
The ranking of interventions for primary outcomes: the incidence rate of moderate-to-severe OHSS. The surface under the cumulative ranking curve values for calcium, hydroxyethyl starch (HES), letrozole, metformin, cabergoline, quinagolide, albumin, aspirin, glucocorticoids and placebo/blank was 92.4, 78.6, 61.3, 58.8, 57.4, 55.7, 41.2 23.0, 18.9 and 12.7%, respectively.

**Figure 4:**
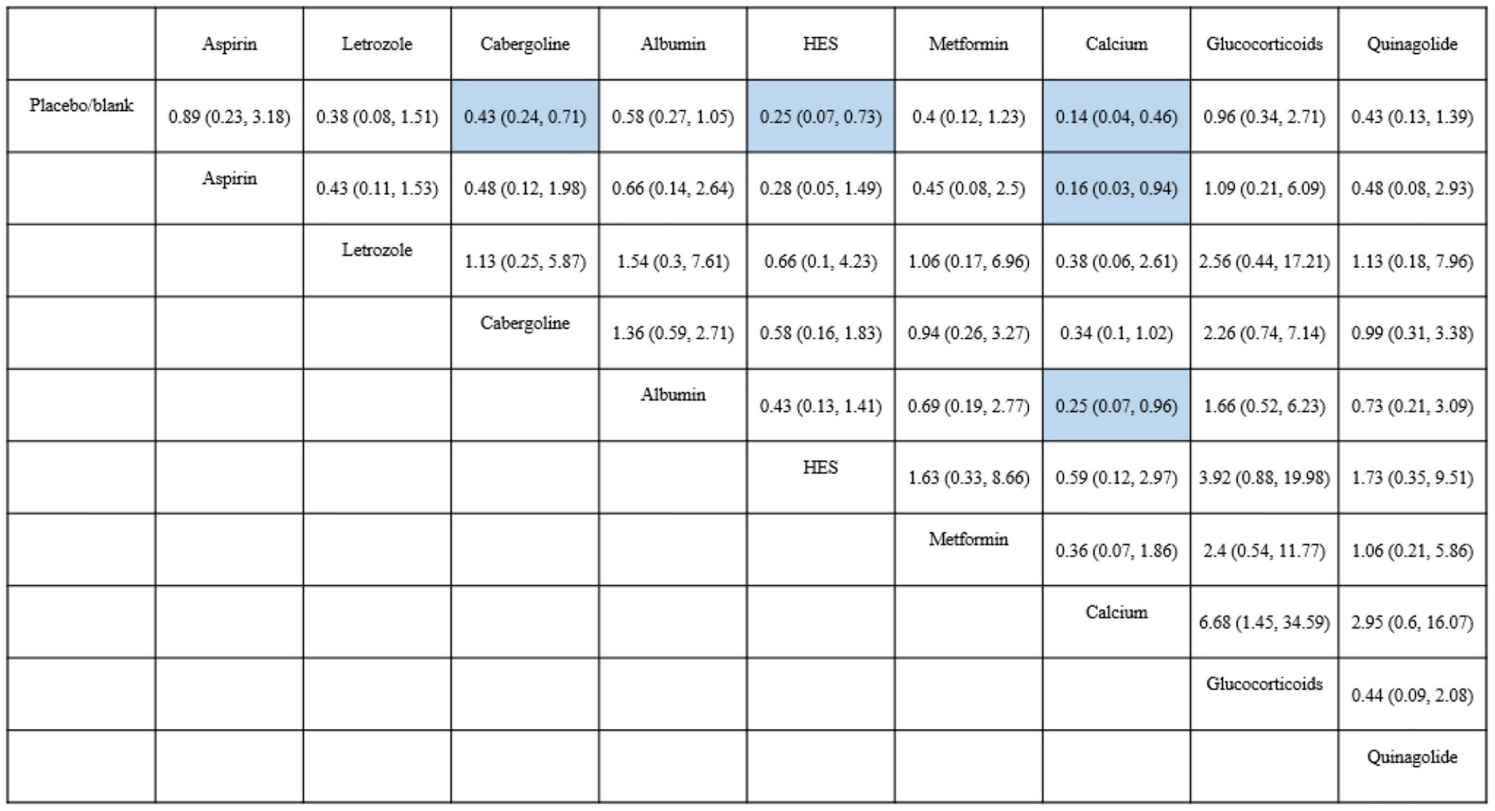
Results of network meta-analysis for moderate-to-severe OHSS. Results are shown as RR (95%CI), representing column-defining treatment versus row-defining treatment. HES, Hydroxyethyl starch. Statistically significant results are shown in blue.

According to the SUCRA results, the rankings in terms of effectiveness in preventing moderate-to-severe OHSS were as follows: calcium (SUCRA, 92.4%), HES (SUCRA, 78.6%), letrozole (SUCRA, 61.3%), metformin (SUCRA, 58.8%), cabergoline (SUCRA, 57.4%), quinagolide (SUCRA, 55.7%), albumin (SUCRA, 41.2%), aspirin (SUCRA, 23.0%), and glucocorticoids (SUCRA, 18.9%) **(** **Figure 3** **)**. The league plot showed that calcium was significantly more effective in preventing OHSS than aspirin and albumin (grade: moderate, moderate, respectively). There was no significant difference among the remaining drugs **(** **Figure 4** **)**. Node splitting revealed inconsistencies in the direct and indirect comparisons between the quinagolide and placebo/blank control groups. As shown in **Supplemental 3.3**, there were no significant inconsistencies between the remaining individual comparisons. There were heterogeneities of 64.7% (I^2^.pair) and 67.3% (I^2^.cons), which represented substantial heterogeneity. The forest plots of each drug were shown in **Supplemental 3.4**.

### Secondary Outcome Measures

#### Clinical Pregnancy Rate

A total of 29 RCTs (n = 3965) were included. The league plot showed that none of the eight drugs affected the clinical pregnancy rate. Albumin significantly reduced clinical pregnancy rates compared with metformin (RR 0.77, 95% CI 0.60, 0.99). There were no significant differences in the other between-drug comparisons **(** **Supplemental 3.5** **).** **Figure 2B** shows the forest plot. There were heterogeneities of 0% (I^2^.pair) and 0% (I^2^.cons), which indicated no heterogeneity.

#### Miscarriage Rate

A total of 18 RCTs (n = 2816) were included. The league plot showed that none of the four drugs (cabergoline, albumin, metformin, and calcium) affected the miscarriage rate. There were no significant differences in the between-drug comparisons **(** **Supplemental 3.6** **)**. **Figure 2C** shows the forest plot. The heterogeneities were 0% (I^2^.pair) and 0% (I^2^.cons), which represented no heterogeneity.

#### Live Birth Rate

A total of 17 RCTs (n = 2616) were included. The league plot showed that none of the four drugs (cabergoline, albumin, metformin, and calcium) affected the live birth rate. There were no significant differences in the between-drug comparisons **(** **Supplemental 3.7** **)**. **Figure 2D** shows the forest plot. The heterogeneities were 35.0% (I^2^.pair) and 22.6% (I^2^.cons), which represented mild heterogeneity.

### Risk of Bias

A few studies did not report allocation concealment and whether double blinding was used; therefore, they were judged as having an unclear risk. The plot of the risk of bias is shown in **Supplemental 3.8**. **Supplemental 3.9** shows a comparison-adjusted funnel plot for the prevention of moderate-to-severe OHSS. Funnel plots for clinical pregnancies, miscarriages, and live births are shown in **Supplemental 3.10-12**. All funnel plots showed good symmetry, with none having publication bias.

## Discussion

There remains inconclusive evidence regarding the best intervention for preventing OHSS given the lack of head-to-head studies for different interventions. This network meta-analysis included 40 RCTs assessing the effectiveness and safety of nine drugs for the prevention of moderate-to-severe OHSS. We found that calcium, HES, and cabergoline significantly reduced the incidence of moderate-to-severe OHSS (grade: high, high, and moderate, respectively). These drugs did not significantly affect the clinical pregnancy rates.

Our network meta-analysis showed that intravenous or oral calcium significantly prevented moderate-to-severe OHSS and was safe for patients. The SUCRA ranking showed that calcium was the most effective intervention for preventing OHSS. One RCT concluded that 200 mL saline containing 10 mL of 10% calcium gluconate for three consecutive days after oocyte retrieval significantly prevented OHSS compared to the placebo group (5). Intravenous or oral calcium was significantly more effective than cabergoline in preventing OHSS (6, 7). Vascular endothelial growth factor (VEGF) is a crucial factor in OHSS development and is associated with follicular growth, luteal function, angiogenesis, and vascular endothelial stimulation (2). The possible underlying mechanism of calcium preventing OHSS could be by inhibiting cyclic adenosine monophosphate-stimulated renin secretion, which reduces the production of angiotensin-converting enzyme II (53–55). This subsequently reduces VEGF expression in human luteinized granulosa cells (53–55).

This network meta-analysis showed that HES significantly prevented moderate-to-severe OHSS and demonstrated good safety. However, HES is less effective than calcium. A previous meta-analysis showed that administering 1000 ml of 6% HES on the day of oocyte retrieval effectively prevented OHSS (56). HES could prevent OHSS due to its large molecular mass of approximately 200,000kDa (14). HES increases the intravascular volume and osmotic pressure as well as reduces blood viscosity (14). Furthermore, HES inhibits platelet aggregation and reduces blood coagulation, which ultimately prevents OHSS development (14).

This network meta-analysis showed that oral administration of 0.5 mg cabergoline for 8 consecutive days starting from the HCG triggering day was safe and effective in preventing moderate-to-severe OHSS. Consistent with previous meta-analyses, cabergoline prevented moderate-to-severe OHSS and did not affect the clinical pregnancy rates, miscarriage rates, live birth rates, or multiple birth rates (57). Cabergoline is a dopamine receptor agonist that selectively binds to dopamine receptors to promote endocytosis of VEGF receptors (58). It blocks VEGF binding, interferes with the VEGF/VEGFR-2 pathway, reduces neovascularization, and decreases vessel permeability, which ultimately prevents OHSS development (58). However, subgroup analysis revealed that cabergoline did not prevent OHSS in women with PCOS. Few studies have investigated OHSS prevention using cabergoline in women with PCOS. Moreover, we found that quinagolide, another dopamine agonist, did not prevent moderate-to-severe OHSS.

Our analysis showed that metformin did not prevent OHSS. Currently, the efficacy of metformin in preventing OHSS remains controversial. The results of this RCT are consistent with our results (37). However, other studies suggest that metformin can prevent OHSS in patients with PCOS (41, 42). Metformin may prevent OHSS by reducing the levels of VEGF, insulin, and E2 on the HCG triggering day (59).

This network meta-analysis showed that letrozole did not prevent OHSS development. The effect of letrozole in preventing OHSS remains unclear. Letrozole is a third-generation aromatase inhibitor used to treat oestrogen receptor-positive breast cancer and as a first-line ovulation induction agent in assisted reproductive technology (60). Mai et al. concluded that using 5.0 mg letrozole daily for 5 days was superior to aspirin in preventing OHSS (24). However, another study showed that using 5.0 mg letrozole daily for 5 days from the day of oocyte retrieval did not prevent OHSS (61). One study compared the use of 7.5 mg and 5.0 mg letrozole daily for preventing moderate-to-severe OHSS (33). They found that 7.5 mg, but not 5.0 mg, per day effectively prevented OHSS (33). However, these findings should be interpreted with caution given the small number of studies and the wide CIs of the results.

Our analysis showed that aspirin did not prevent OHSS or PCOS, which is consistent with the findings of Namavar et al. (25). Consistent with a previous meta-analysis, albumin did not prevent OHSS (62). There are inconsistent reports regarding the role of glucocorticoids in preventing OHSS (10, 11, 63). Our analysis showed that glucocorticoids did not prevent OHSS. However, Revelli et al. found that acetylsalicylic acid combined with glucocorticoids could prevent severe OHSS and increase the number of oocytes retrieved (63).

This network meta-analysis has several advantages. It analysed the effects of nine drugs on the prevention of OHSS, clarified that three drugs (calcium, HES, and cabergoline) were able to prevent OHSS, and to the best of our knowledge, is the first of its kind to compare the effectiveness of different drugs. The effects of eight drugs (except letrozole) on clinical pregnancy rates were also analysed. It was conducted in strict accordance with the recommendations of the Cochrane Handbook and PRISMA statement. Furthermore, we conducted a comprehensive search of different databases to extract all relevant RCTs. However, this study has several limitations. First, there were missing direct comparative results between some interventions, which could have led to bias in the study results even though consistency models were used for fitting. Given the large number of studies that did not report the BMI, we could not conduct subgroup analysis based on the BMI. The SUCRA curve was used to estimate the ranking of effectiveness between the different interventions; however, it has limitations and the results should be interpreted with caution. The included RCTs reported few miscarriages and live births.

## Conclusions

In conclusion, calcium, HES, and cabergoline are safe and effective in preventing moderate-to-severe OHSS. SUCRA showed that calcium is the most effective intervention for preventing moderate-to-severe OHSS. Given the limitations of this study, the aforementioned conclusions should be validated by large-scale multi-centre RCTs. Determining the effectiveness of various drugs for OHSS prevention could facilitate the establishment of the best protocol for OHSS prevention.

## Data Availability Statement

The original contributions presented in the study are included in the article/supplementary material. Further inquiries can be directed to the corresponding author.

## Author Contributions

JZ, DW, and HS were the principal investigators. They formulated the meta-analysis and wrote the manuscript. DW and JZ contributed to the acquisition of the data and the manuscript writing process. TY helped in the acquisition of the data. YY created the statistical analysis. All authors also contributed to the critical revision of the intellectual content and approved the final version of the paper.

## Funding

This work was supported by the National Natural Science Foundation of China (Grant No. 82071649) and the Key Scientific Research Projects of Higher Education Institutions in Henan Province (Grant No. 22A320025).

## Conflict of Interest

The authors declare that the research was conducted in the absence of any commercial or financial relationships that could be construed as a potential conflict of interest.

## Publisher’s Note

All claims expressed in this article are solely those of the authors and do not necessarily represent those of their affiliated organizations, or those of the publisher, the editors and the reviewers. Any product that may be evaluated in this article, or claim that may be made by its manufacturer, is not guaranteed or endorsed by the publisher.
