# Supplementary material for: Comparison of the Effectiveness of Various Medicines in the Prevention of Ovarian Hyperstimulation Syndrome: A Network Meta-Analysis of Randomized Controlled Trials
Source: Front Endocrinol (Lausanne). 2022 Jan 26;13:808517. doi: 10.3389/fendo.2022.808517 (PMC8825486; doi:10.3389/fendo.2022.808517)
Supplement: Supplementary file 1 [file DataSheet_1.docx]

PubMed, Embase, Web of Science and the Cochrane Library

PUBMED search strategy Database: https://www.ncbi.nlm.nih.gov/pubmed

#1 "Aspirin"[Mesh]

#2 (((letrozol*) OR anastrazol*)) OR "Aromatase Inhibitors"[Mesh]

#3 “Albumin”

#4 “Metformin” [Mesh]

#5  "Calcium"[Mesh] OR "Ca"

#6 "Cabergoline"[Mesh]

#7 "Quinagolide"

#8 "Hydroxyethyl starch"

#9  (Glucocorticoid*) OR (Prednisolone*)

#10 “Bromocriptine”[Mesh]

#11 “Progesterone”[Mesh]

#12 #1 OR #2 OR #3 OR #4 OR #5 OR #6 OR #7 OR #8 OR #9 OR #10 OR #11

#13 (Prevent*) OR (reduce*) OR (decrease*)

#14 (((((((in vitro fertilization) OR in-vitro fertilization) OR IVF) OR ICSI) OR intra-cytoplasmic sperm injection) OR intracytoplasmic sperm injection)) OR "Fertilization in Vitro"[Mesh]

#15 randomized controlled trial[Publication Type] OR (random*) [Title/Abstract] OR placebo[Title/Abstract]

#16 (OHSS) OR “ovarian hyperstimulation syndrome” [Mesh]

#17 #13 AND #14 AND #15 AND #16

Embase search strategy Database: EMBASE.com

#1 aspirin:ti,ab,kw

#2 letrozol*:ti,ab,kw OR anastrazol*:ti,ab,kw OR 'aromatase inhibitors':ti,ab,kw

#3 'albumin':ti,ab,kw

#4 'metformin':ti,ab,kw

#5 'calcium':ti,ab,kw OR 'ca':ti,ab,kw

#6 'cabergoline':ti,ab,kw

#7 'quinagolide':ti,ab,kw

#8 'hydroxyethyl starch':ti,ab,kw

#9 glucocorticoid*:ti,ab,kw OR prednisolone*:ti,ab,kw

#10 'bromocriptine':ti,ab,kw

#11 'progesterone':ti,ab,kw

#12 #1 OR #2 OR #3 OR #4 OR #5 OR #6 OR #7 OR #8 OR #9 OR #10 OR #11

#13 prevent* OR reduce* OR decrease*

#14 (((in AND vitro AND fertilization OR 'in vitro') AND fertilization OR ivf OR icsi OR 'intracytoplasmic') AND sperm AND injection OR intracytoplasmic) AND sperm AND injection OR 'fertilization in vitro'

#15 'randomized controlled trial':it OR random*

#16 ohss OR 'ovarian hyperstimulation syndrome'

#17 #13 AND #14 AND #15 AND #16

Web of science

#1 TS=(Aspirin)

#2 TS=((((letrozol*) OR anastrazol*)) OR "Aromatase Inhibitors")

#3 TS=(Albumin)

#4 TS=(Metformin)

#5 TS=(Calcium)

#6 TS=(Cabergoline)

#7 TS=(Quinagolide)

#8 TS=(Hydroxyethyl starch)

#9 TS=((Glucocorticoid*) OR (Prednisolone*))

#10 TS=(Bromocriptine)

#11 TS=(Progesterone)

#12 (#1 OR #2 OR #3 OR #4 OR #5 OR #6 OR #7 OR #8 OR #9 OR #10 OR #11)

#13 TS=((Prevent*) OR (reduce*) OR (decrease*))

#14 TS=((((((((in vitro fertilization) OR in-vitro fertilization) OR IVF) OR ICSI) OR intra-cytoplasmic sperm injection) OR intracytoplasmic sperm injection)) OR "Fertilization in Vitro")

#15 TS=( randomized controlled trial OR (random*) OR placebo)

#16 TS=((OHSS) OR “ovarian hyperstimulation syndrome”)

#17 #13 AND #14 AND #15 AND #16

Cochrane Library

#1 Aspirin

#2 (((letrozol*) OR anastrazol*)) OR "Aromatase Inhibitors"

#3 Albumin

#4 Metformin

#5 Calcium

#6 Cabergoline

#7 Quinagolide

#8 Hydroxyethyl starch

#9 (Glucocorticoid*) OR (Prednisolone*)

#10 Bromocriptine

#11 Progesterone

#12 #1 OR #2 OR #3 OR #4 OR #5 OR #6 OR #7 OR #8 OR #9 OR #10 OR #11

#13 (Prevent*) OR (reduce*) OR (decrease*)

#14 (((((((in vitro fertilization) OR in-vitro fertilization) OR IVF) OR ICSI) OR intra-cytoplasmic sperm injection) OR intracytoplasmic sperm injection)) OR "Fertilization in Vitro"

#15 randomized controlled trial OR (random*) OR placebo

#16 (OHSS) OR “ovarian hyperstimulation syndrome”

#17 #13 AND #14 AND #15 AND #16
