# Supplementary material for: Comparison of the Effectiveness of Various Medicines in the Prevention of Ovarian Hyperstimulation Syndrome: A Network Meta-Analysis of Randomized Controlled Trials
Source: Front Endocrinol (Lausanne). 2022 Jan 26;13:808517. doi: 10.3389/fendo.2022.808517 (PMC8825486; doi:10.3389/fendo.2022.808517)
Supplement: Supplementary file 2 [file DataSheet_2.docx]

1. El-Khayat W, Elsadek M. Calcium infusion for the prevention of ovarian hyperstimulation syndrome: a double-blind randomized controlled trial. Fertility and sterility 2015;103:101-5.
2. Elnory MA, Elmantwe ANM. Comparison of cabergoline versus calcium infusion in ovarian hyperstimulation syndrome prevention: A randomized clinical trial. Middle East Fertility Society Journal 2018;23:357-62.
3. Samy Saad A AAMK. Calcium dobesilate versus cabergoline for prevention of ovarian hyper stimulation syndrome. Reproductive System & Sexual Disorders: Current Research 2017;6:1-5.
4. Naredi N, Karunakaran S. Calcium gluconate infusion is as effective as the vascular endothelial growth factor antagonist cabergoline for the prevention of ovarian hyperstimulation syndrome. Journal of human reproductive sciences 2013;6:248-52.
5. Fouda UM, Elshaer HS, Youssef GG, Hanafy A, Mehrem WM, Youssef MA et al. Cabergoline versus calcium infusion in the prevention of ovarian hyperstimulation syndrome: a randomised controlled study. Journal of Obstetrics and Gynaecology 2021;1-5.
6. Mohammadi Yeganeh L, Moini A, Shiva M, Mirghavam N, Bagheri Lankarani N. Methylprednisolone for prevention of ovarian hyperstimulation syndrome in patients with polycystic ovarian syndrome undergoing in-vitro fertilisation: a randomised controlled trial. Journal of obstetrics and gynaecology : the journal of the Institute of Obstetrics and Gynaecology 2018;38:241-6.
7. Tan SL, Balen A, el Hussein E, Campbell S, Jacobs HS. The administration of glucocorticoids for the prevention of ovarian hyperstimulation syndrome in in vitro fertilization: a prospective randomized study. Fertility and sterility 1992;58:378-83.
8. Salah AM, El-Helew Y. Can cabergoline prevent ovarian hyperstimulation syndrome in polycystic ovarian patients undergoing gonadotropin stimulation? Evidence Based Womenʼs Health Journal 2012;2:56-9.
9. Gokmen O UM, Ekin M, Keles G, Turan C, Oral H. Intravenous albumin versus hydroxyethyl starch for the prevention of ovarian hyperstimulation in an in-vitro fertilization programme a prospective randomized placebo controlled study. Eur J Obstet Gynecol Reprod Biol 2001;96:187-92.
10. König E, Bussen S, Sütterlin M, Steck T. Prophylactic intravenous hydroxyethyle starch solution prevents moderate-severe ovarian hyperstimulation in in-vitro fertilization patients: a prospective, randomized, double-blind and placebo-controlled study. Human reproduction (Oxford, England) 1998;13(9):2421-2424.
11. Tehraninejad ES, Hafezi M, Arabipoor A, Aziminekoo E, Chehrazi M, Bahmanabadi A. Comparison of cabergoline and intravenous albumin in the prevention of ovarian hyperstimulation syndrome: a randomized clinical trial. Journal of assisted reproduction and genetics 2012;29:259-64.
12. Shalev E GY, Matilsky M, Ben-Ami M. Decreased incidence of severe ovarian hyperstimulation syndrome in high risk in-vitro fertilization patients receiving intravenous albumin a prospective study. Human reproduction (Oxford, England) 1995;10(6):1373-6.
13. Isikoglu M, Berkkanoglu M, Senturk Z, Ozgur K. Human albumin does not prevent ovarian hyperstimulation syndrome in assisted reproductive technology program: a prospective randomized placebo-controlled double blind study. Fertility and sterility 2007;88:982-5.
14. Bellver J, Muñoz EA, Ballesteros A, Soares SR, Bosch E, Simón C et al. Intravenous albumin does not prevent moderate-severe ovarian hyperstimulation syndrome in high-risk IVF patients: a randomized controlled study. Human reproduction (Oxford, England) 2003;18:2283-8.
15. Shoham Z WA, Barash A, Borenstein R, Schachter M, Insler V. Intravenous albumin for the prevention of severe ovarian hyperstimulation syndrome in an in vitro fertilization program a prospective, randomized, placebo-controlled study. Fertil Steril 1994;62(1):137-42.
16. Isik AZ GO, Zeyneloglu HB, Kara S, Keles G, Gulekli B. Intravenous albumin prevents moderate-severe ovarian hyperstimulation in in-vitro fertilization patients a prospective, randomized and controlled study. Eur J Obstet Gynecol Reprod Biol 1996;70(2):179-83.
17. Ben-Chetrit A E-GT, Gal M, Huerta M, Mimon T, Algur N, Diamant YZ, Margalioth EJ. The questionable use of albumin for the prevention of ovarian hyperstimulation syndrome in an IVF programme a randomized placebo-controlled trial. Hum Reprod 2001;16(9):1880-4.
18. Torabizadeh A VF, Ghorbanpour Z. Comparison of albumin and cabergoline in the prevention of ovarian hyperstimulation syndrome a clinical trial study. Iran J Reprod Med 2013;11(10):837-42.
19. Ghahiri A MN, Movahedi M, Hosseini N. Evaluation of intravenous hydroxylethyl starch, intravenous albumin 20%, and oral cabergoline for prevention of ovarian hyperstimulation syndrome in patients undergoing ovulation in patients undergoing ovulation induction. Journal of research in medical sciences : the official journal of Isfahan University of Medical Sciences 2015;20(7):692-6.
20. Mai Q, Hu X, Yang G, Luo Y, Huang K, Yuan Y et al. Effect of letrozole on moderate and severe early-onset ovarian hyperstimulation syndrome in high-risk women: a prospective randomized trial. American journal of obstetrics and gynecology 2017;216(1):42.e1-42.e10.
21. Namavar Jahromi B, Zolghadri J, Rahmani E, Alipour S, Anvar Z, Zarei A et al. Effect of low-dose aspirin on the development of ovarian hyperstimulation syndrome and outcomes of assisted reproductive techniques in the women with PCOS, a randomized double-blinded clinical trial. Taiwanese journal of obstetrics & gynecology 2019;58:255-60.
22. Carizza C AV, Abdelmassih S, Ravizzini P, Salgueiro L, Salgueiro PT, Jine LT, Nagy P, Abdelmassih R. Cabergoline reduces the early onset of ovarian hyperstimulation syndrome a prospective randomized study. Reproductive biomedicine online 2008;17(6):751-5.
23. Shaltout A, Shohyab A, Youssef MA. Can dopamine agonist at a low dose reduce ovarian hyperstimulation syndrome in women at risk undergoing ICSI treatment cycles? A randomized controlled study. European journal of obstetrics, gynecology, and reproductive biology 2012;165:254-8.
24. Jellad S, Haj Hassine A, Basly M, Mrabet A, Chibani M, Rachdi R. Vascular endothelial growth factor antagonist reduces the early onset and the severity of ovarian hyperstimulation syndrome. Journal of gynecology obstetrics and human reproduction 2017;46:87-91.
25. Alvarez C, Martí-Bonmatí L, Novella-Maestre E, Sanz R, Gómez R, Fernández-Sánchez M et al. Dopamine agonist cabergoline reduces hemoconcentration and ascites in hyperstimulated women undergoing assisted reproduction. The Journal of clinical endocrinology and metabolism 2007;92:2931-7.
26. Amir H YD, Hasson J, Amit A, Gordon D, Azem F. Cabergoline for reducing ovarian hyperstimulation syndrome in assisted reproductive technology treatment cycles. A prospective randomized controlled trial. J Reprod Med 2015;60(1-2):48-54.
27. Taheripanah R, Vasef M, Zamaniyan M, Taheripanah A. Comparison of Cabergoline and Quinagolide in Prevention of Severe Ovarian Hyperstimulation Syndrome among Patients Undergoing Intracytoplasmic Sperm Injection. International journal of fertility & sterility 2018;12:1-5.
28. Kilic N, Ozdemir O, Basar HC, Demircan F, Ekmez F, Yucel O. Cabergoline for preventing ovarian hyperstimulation syndrome in women at risk undergoing in vitro fertilization/intracytoplasmic sperm injection treatment cycles: A randomized controlled study. Avicenna journal of medicine 2015;5:123-7.
29. He Q, Liang L, Zhang C, Li H, Ge Z, Wang L et al. Effects of different doses of letrozole on the incidence of early-onset ovarian hyperstimulation syndrome after oocyte retrieval. Systems biology in reproductive medicine 2014;60:355-60.
30. Swanton A, Lighten A, Granne I, McVeigh E, Lavery S, Trew G et al. Do women with ovaries of polycystic morphology without any other features of PCOS benefit from short-term metformin co-treatment during IVF? A double-blind, placebo-controlled, randomized trial. Human reproduction (Oxford, England) 2011;26:2178-84.
31. Abdalmageed OS, Farghaly TA, Abdelaleem AA, Abdelmagied AE, Ali MK, Abbas AM. Impact of Metformin on IVF Outcomes in Overweight and Obese Women With Polycystic Ovary Syndrome: A Randomized Double-Blind Controlled Trial. Reproductive sciences (Thousand Oaks, Calif) 2019;26:1336-42.
32. Qublan HS, Al-Khaderei S, Abu-Salem AN, Al-Zpoon A, Al-Khateeb M, Al-Ibrahim N et al. Metformin in the treatment of clomiphene citrate-resistant women with polycystic ovary syndrome undergoing in vitro fertilisation treatment: A randomised controlled trial. Journal of Obstetrics and Gynaecology 2009;29:651-5.
33. Palomba S, Falbo A, Carrillo L, Villani MT, Orio F, Russo T et al. Metformin reduces risk of ovarian hyperstimulation syndrome in patients with polycystic ovary syndrome during gonadotropin-stimulated in vitro fertilization cycles: a randomized, controlled trial. Fertility and sterility 2011;96:1384-90.e4.
34. Kjotrod SB, von During V, Carlsen SM. Metformin treatment before IVF/ICSI in women with polycystic ovary syndrome; a prospective, randomized, double blind study. Human Reproduction 2004;19:1315-22.
35. Onalan G, Pabuccu R, Goktolga U, Ceyhan T, Bagis T, Cincik M. Metformin treatment in patients with polycystic ovary syndrome undergoing in vitro fertilization: a prospective randomized trial. Fertility and sterility 2005;84:798-801.
36. Cheraghi E, Mehranjani MS, Shariatzadeh MA, Esfahani MH, Ebrahimi Z. N-Acetylcysteine improves oocyte and embryo quality in polycystic ovary syndrome patients undergoing intracytoplasmic sperm injection: an alternative to metformin. Reprod Fertil Dev 2016;28:723-31.
37. An Y, Sun Z, Zhang Y, Liu B, Guan Y, Lu M. The use of berberine for women with polycystic ovary syndrome undergoing IVF treatment. Clinical endocrinology 2014;80:425-31.
38. Tang T, Glanville J, Orsi N, Barth JH, Balen AH. The use of metformin for women with PCOS undergoing IVF treatment. Human reproduction (Oxford, England) 2006;21:1416-25.
39. Kjotrod SB, Carlsen SM, Rasmussen PE, Holst-Larsen T, Mellembakken J, Thurin-Kjellberg A et al. Use of metformin before and during assisted reproductive technology in non-obese young infertile women with polycystic ovary syndrome: a prospective, randomized, double-blind, multi-centre study. Human reproduction (Oxford, England) 2011;26:2045-53.
40. Busso C, Fernández-Sánchez M, García-Velasco JA, Landeras J, Ballesteros A, Muñoz E et al. The non-ergot derived dopamine agonist quinagolide in prevention of early ovarian hyperstimulation syndrome in IVF patients: a randomized, double-blind, placebo-controlled trial. Human reproduction (Oxford, England) 2010;25:995-1004.
