# Supplementary material for: Comparison of the Effectiveness of Various Medicines in the Prevention of Ovarian Hyperstimulation Syndrome: A Network Meta-Analysis of Randomized Controlled Trials
Source: Front Endocrinol (Lausanne). 2022 Jan 26;13:808517. doi: 10.3389/fendo.2022.808517 (PMC8825486; doi:10.3389/fendo.2022.808517)
Supplement: Supplementary file 3 [file DataSheet_3.docx]

Content

Supplemental 3.1 OHSS classification.

Supplemental 3.2 Network plot of moderate-to-severe OHSS.

Supplemental 3.3 Inconsistency plot.

Supplemental 3.4 Forest plots of each medicine.

Supplemental 3.5 League plot for clinical pregnancy rate.

Supplemental 3.6 League plot for miscarriage rate.

Supplemental 3.7 League plot for live birth rate.

Supplemental 3.8 Risk of bias

Supplemental 3.9 Comparison-adjusted funnel plot for moderate-to-severe OHSS.

Supplemental 3.10 Comparison-adjusted funnel plot for clinical pregnancy rate.

Supplemental 3.11 Comparison-adjusted funnel plot for miscarriage rate.

Supplemental 3.12 Comparison-adjusted funnel plot for live birth rate.

Supplemental 3.1 OHSS classification

| **OHSS criteria** | **Classification** |
| --- | --- |
| Navot, 1992 | Mild:Abdominal bloating/discomfort; Mild nausea/vomiting; Diarrhea; Enlarged ovaries; No important laboratory alterations;  Moderate: Mild features +; Elevated hematocrit (>41 %); Ultrasonographic evidence of ascites; Elevated WBC (>15*10 9 /L); Hypoproteinemia Severe: Mild and moderate features +; Hemoconcentration (hematocrit >55 %); Clinical evidence of ascites; WBC >25*10 9 /L; Hydrothorax; Creatinine clearance <50 mL/min; Severe dyspnea Creatinine >115µmol/L; Oliguria/anuria Na+ <135 mmol/L; Intractable nausea/vomiting; K+ >5.0 mmol/L; Tense ascites; Elevated liver enzymes; Low blood/central venous pressure; Rapid weight gain (>1 kg in 24 hours);  Syncope; Severe abdominal pain; Venous thrombosis Critical: Anuria/acute renal failure; Worsening of findings; Arrhythmia;  Thromboembolism; Pericardial effusion; Large pleural effusion; Arterial thrombosis; Adult respiratory distress syndrome |
| Garder, 2006; Aubuchon,2012 | The cases of mild OHSS with abdominal distention, bloating and ovaries ≤5 cm are observed in a large number of ART cycles without clinical significance and were not included among the OHSS cases in this study. Moderate OHSS is diagnosed with abdominal pain, ultrasonographic measurements of ovarian sizes between 5 and 12 cm and any amount of ascites. Severe OHSS is diagnosed when ascites is clinically detectable with marked abdominal discomfort, liver dysfunction, dyspnea, hypotension, oliguria, hyponatremia and hyperkalemia.  Critical grades are diagnosed when end-organ morbidities like acute respiratory distress syndrome, thromboembolic complications or renal failure are identified and the patients need Intensive care unit (ICU) admission |
| Golan, 1989 | Mild:Grade1.Only laboratory evidence of OHSS exists: total urinary estrogens above 150μg/24 hr and pregnadiol excretion above 10mg/24hr. Grade2.The features of grade 1 plus enlargement of the ovaries. Mild OHSS is, in fact, in common feature of ovulation induction. Ovaries in this category are not enlarged beyond 5cm in diameter. Nothing more than rest and observation is needed for the management of grade2 OHSS. Moderate: Grade3. The features of mild OHSS plus abdominal distension. Grade4. The features of grade3 plus nause, vomiting, and/or diarrhea. Severe: Grade5. The features of moderate OHSS plus ascites and/or hydrothorax. Grade6. All of the above plus a change in blood volume and increased blood viscosity due to hemo-concentration, resulting in coagulation abnormalities and diminished renal perfusion complicated by anuria and renal failure. |
| Schenker and Weinstein,1978 | Mild: Hyperstimulation Grade1. This grading consists only of laboratory findings of hyperstimulation: estrogen levels above 150 J.tg/24 hours and pregnanediol excretion above 10 mg/24 hours. Grade 2. The above laboratory findings are present plus enlargement of ovaries; sometimes small cysts are palpable. Moderate: Hyperstimulation Grade3. In addition to elevated urinary steroid levels and ovarian cysts, abdominal distention is present. Grade4. This grading consists of criteria of grade 3, plus vomiting and/or diarrhea. Severe: Hyperstimulation Grade5. In addition to the above, the ovarian cysts are large and ascites and/or hydrothorax are present. Grade6. Marked hemoconcentration with increased blood viscosity may result in coagulation abnormalities. |
| RCOG, 2006 | Mild: Abdominal bloating; Mild abdominal pain; Ovarian size usually＜8cm2 Moderate: Moderate abdominal pain; Nausea±vomiting; Ultrasound evidence of ascites; Ovarian size usually 8-12cm Severe: Clinical ascites (±hydrothorax); Oliguria (＜300 ml/day or ＜30ml/hour); Haematocrit＞0.45; Hyponatraemia (sodium＜135 mmol/l); Hypo-osmolality (osmolality＜282 mOsm/kg); Hyperkalaemia (potassium＞5 mmol/l); Hypoproteinaemin (serum album ＜35 g/l); Ovarian size usually ＞12cm Critical OHSS: Tense ascites/large hydrothorax; Haematocrit＞0.55; White cell count＞25000/ml; Oliguria/anuria; Thromboembolism; Acute respiratory diatress syndrome |
| Mathur, 2005 | Mild: Abdominal bloating; Mild abdominal pain; Ovarian size usually＜8cm2 Moderate: Moderate abdominal pain; Nausea±vomiting; Ultrasound evidence of ascites; Ovarian size usually 8-12cm Severe: Clinical ascites (±hydrothorax); Oliguria; Haemoconcentration; Hypoproteinaemia; Ovarian size usually＞12cm; Complications: Adult respiratory distress syndrome; Renal failure; Thrombo-embolic phenomena. |
| Humaidan, 2010 | Mild:Fluid in Douglas pouch; Abdominal distention; Pelvic discomfort; Breathing disorder(±); Acute pain(±); Nausea/vomiting(±); Ovarian enlargement; Pregnancy occurrence(±) Moderate: Fluid around uterus; Hematocrit＞45%; White blood cells＞15000/mm3(±); Low urine output(±);Creatinine＞1.5mg/dL(±); Elevated transaminases(±) Severe: White blood cells＞15000/mm3; Low urine output; Clotting disorder(±); Pleural effusion(±); Pregnancy occurrence |
| Aboulghar, 2003 | Moderate: Discomfort, pain, nause, distension, ultrasonic evidence of ascites and enlarged ovaries, normal haematological and biological profiles Severe: Grade A: Dyspnoea, oliguria, nausea, vomiting, diarrhoea, abdominal pain, clinical evidence of ascites, marked distension of abdomen or hydrothorax, US showing large ovaries and marked ascites, normal biochemical profile Grade B: Grade A plus massive tension ascites, markedly enlarged ovaries, severe dyspnoea and marked oliguria, increased haematocrit, elevated serum creatinine and liver dysfunction Grade C: Complications as respiratory distress syndrome, renal shut-down or venous thrombosis |


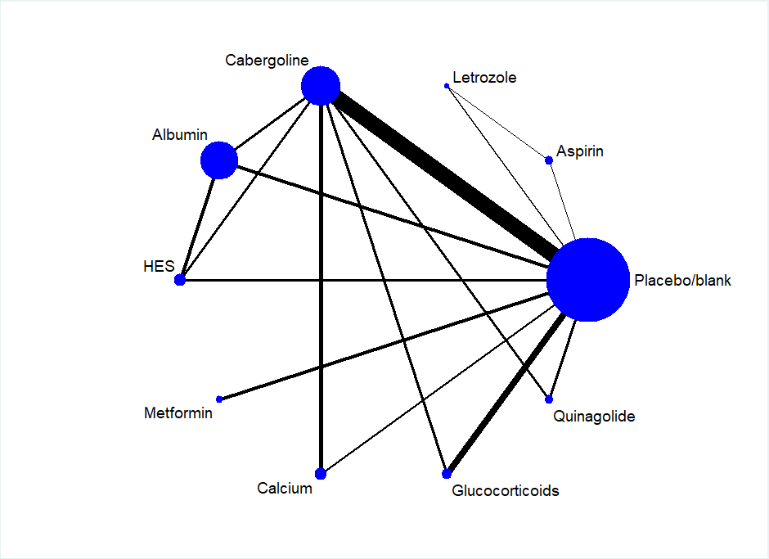


Supplementary 3.2 Network plot of moderate-to-severe OHSS

Network plot showing direct evidence among the compared treatments, in which the size of the circle represents the sample size and the thickness of the line represents the number of studies. HES: hydroxyethyl starch.


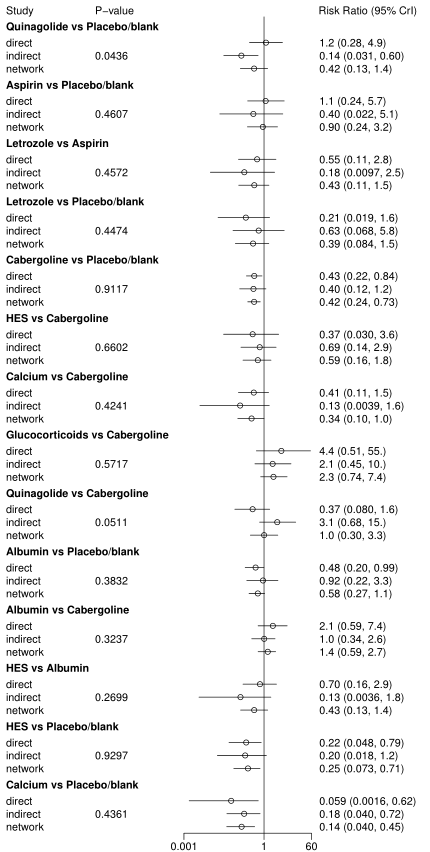


Supplemental 3.3 Inconsistency plot

It presents the results of direct and indirect comparisons (RR, 95%CI). P value less than 0.05 indicates that the difference is statistically significant.


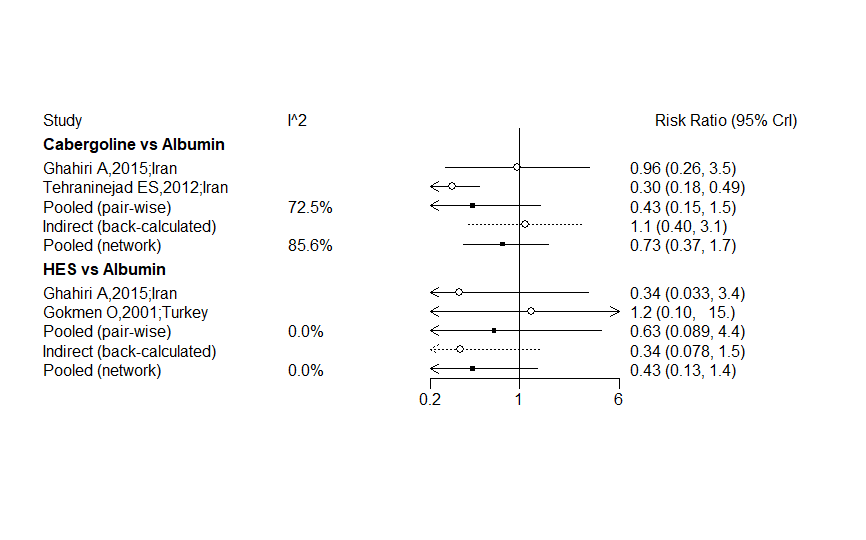


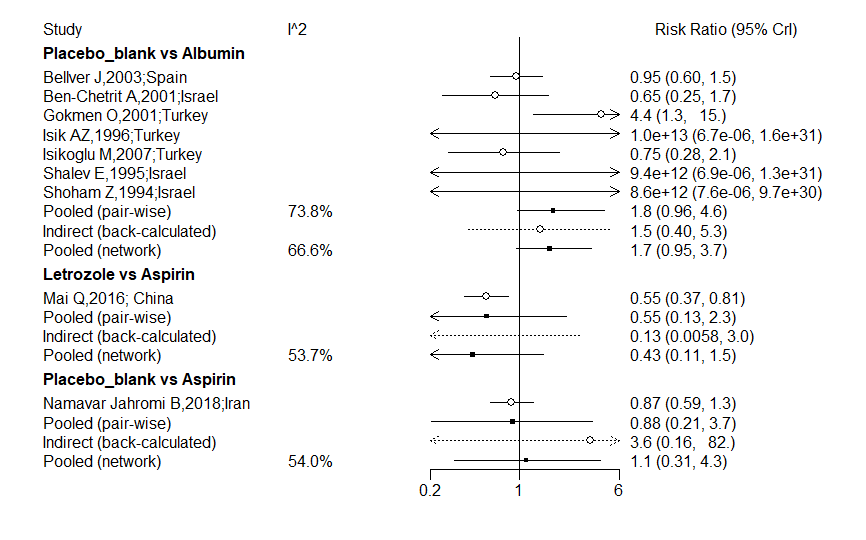


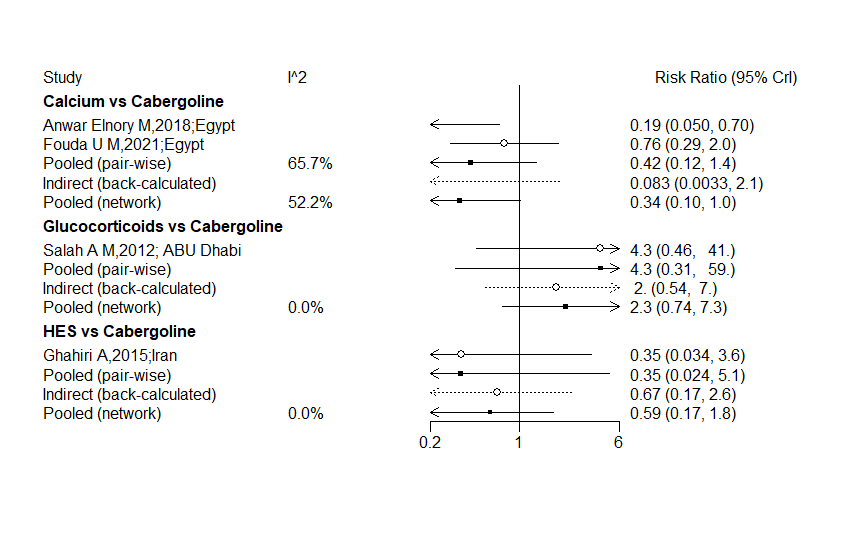


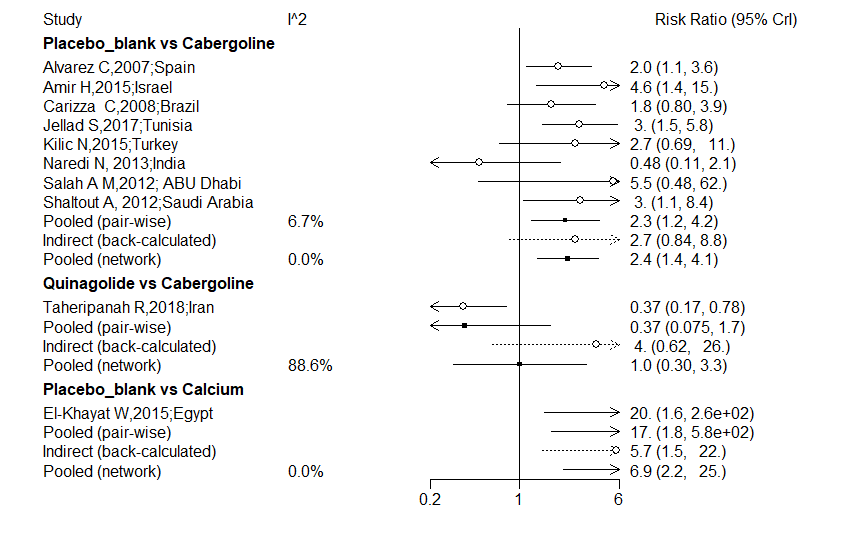


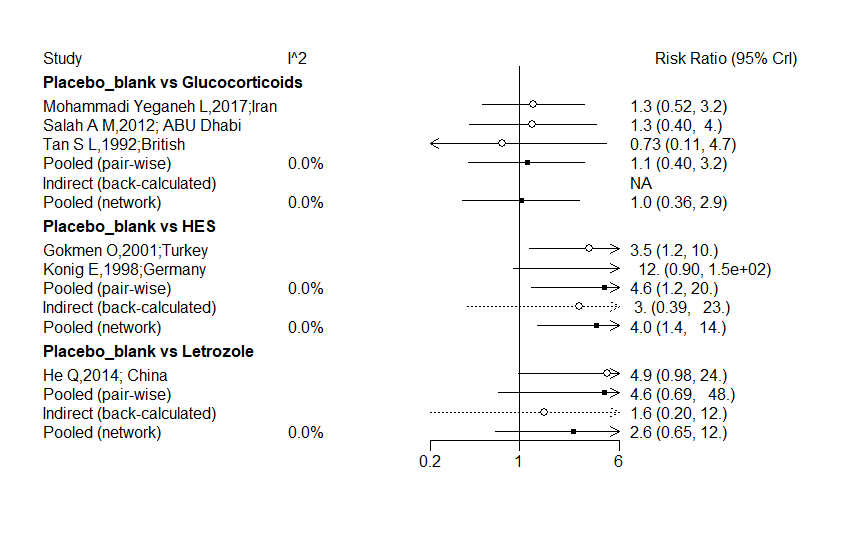


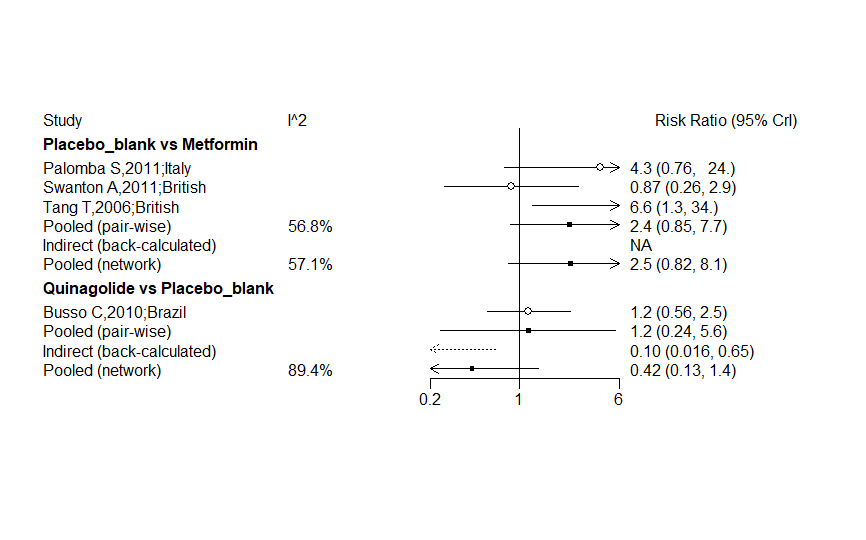


Supplemental 3.4 Forest plots of each medicine.


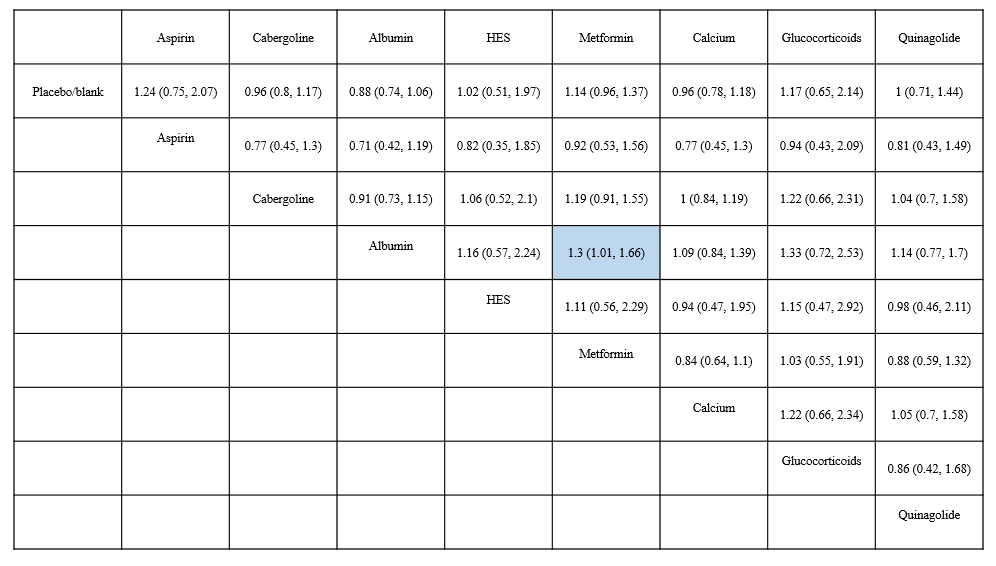


Supplemental 3.5 League plot for clinical pregnancy rate.

Results of network meta-analysis for clinical pregnancy rate. Results are shown as RR (95%CI), representing column-defining treatment versus row-defining treatment. HES: Hydroxyethyl starch. Statistically significant results are shown in blue.


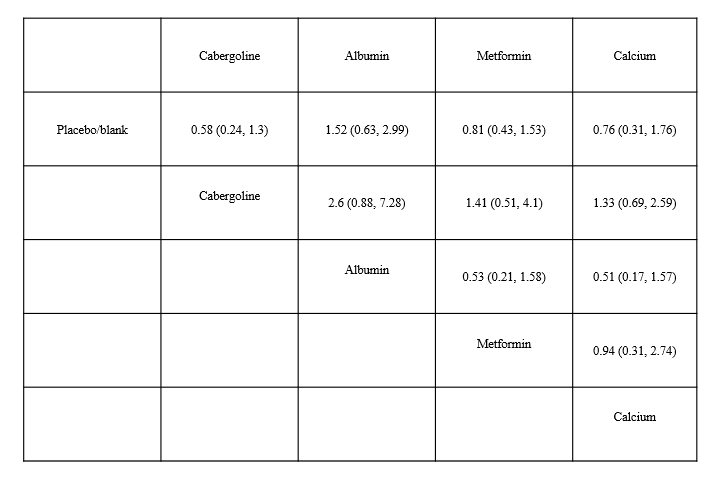


Supplemental 3.6 League plot for miscarriage rate.

Results of network meta-analysis for miscarriage rate. Results are shown as RR (95%CI), representing column-defining treatment versus row-defining treatment. Statistically significant results are shown in blue.


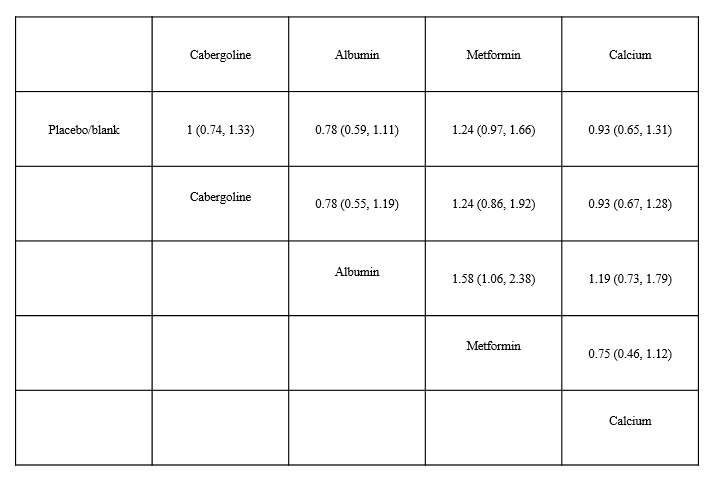


Supplemental 3.7 League plot for live birth rate.

Results of network meta-analysis for live birth rate. Results are shown as RR (95%CI), representing column-defining treatment versus row-defining treatment. Statistically significant results are shown in blue.


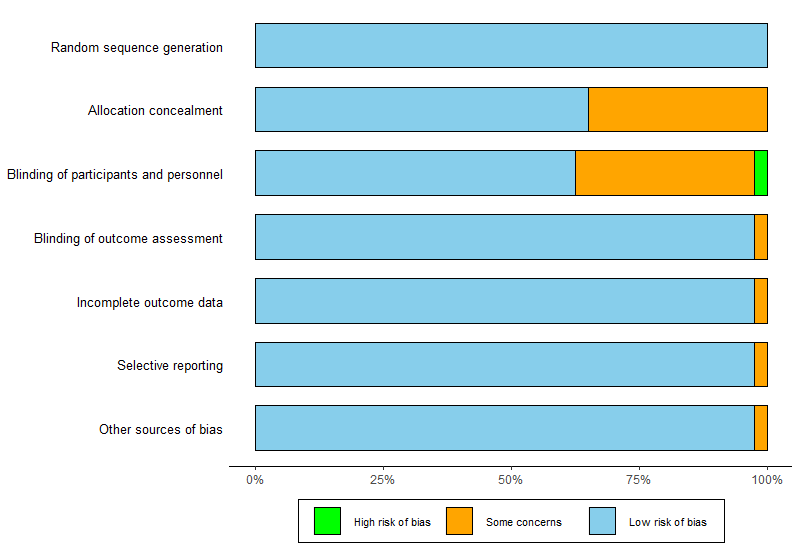


Supplemental 3.8 Risk of bias


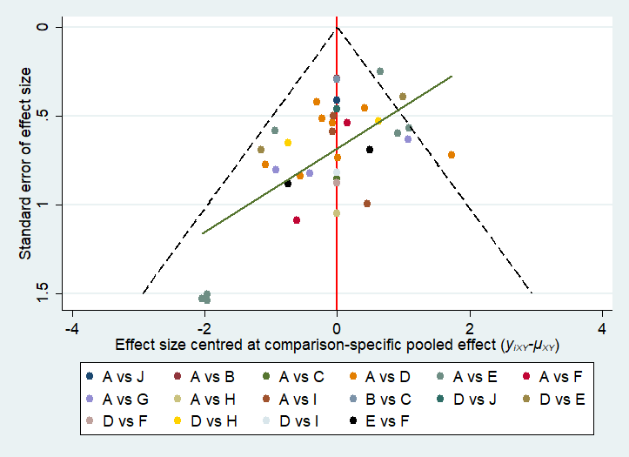


Supplemental 3.9 Comparison-adjusted funnel plot for moderate-to-severe OHSS.

A: Placebo/blank, B: Aspirin, C: Letrozole, D: Cabergoline, E: Albumin, F: Hydroxyethyl starch, G: Metformin, H: Calcium, I: Glucocorticoids, J: Quinagolide.


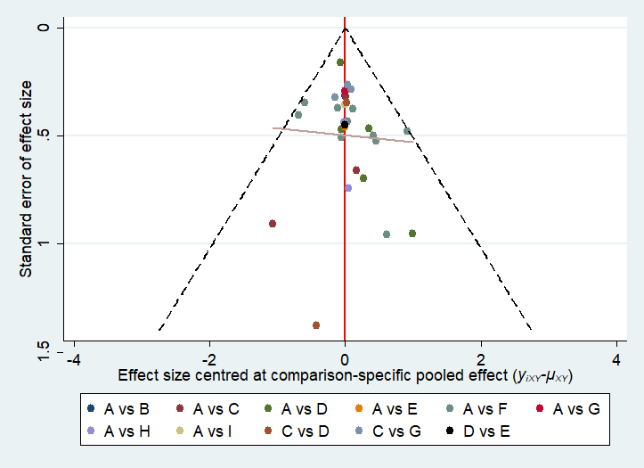


Supplemental 3.10 Comparison-adjusted funnel plot for clinical pregnancy rate.

A: Placebo/blank, B: Aspirin, C: Cabergoline, D: Albumin, E: Hydroxyethyl starch, F: Metformin, G: Calcium, H: Glucocorticoids, I:Quinagolide.


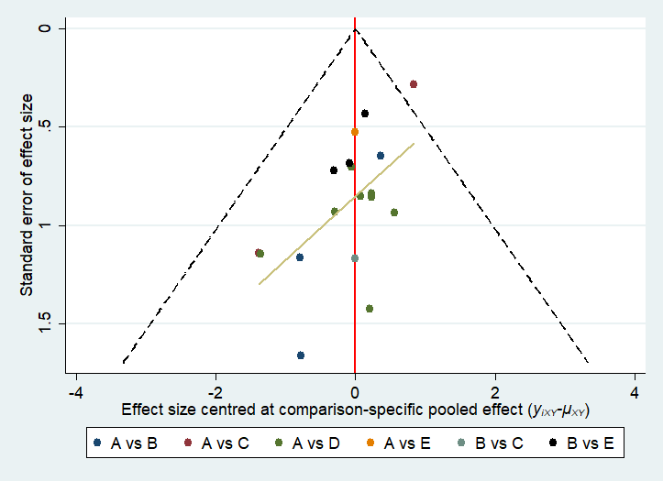


Supplemental 3.11 Comparison-adjusted funnel plot for miscarriage rate.

A: Placebo/blank, B: Cabergoline, C: Albumin, D: Metformin, E: Calcium.


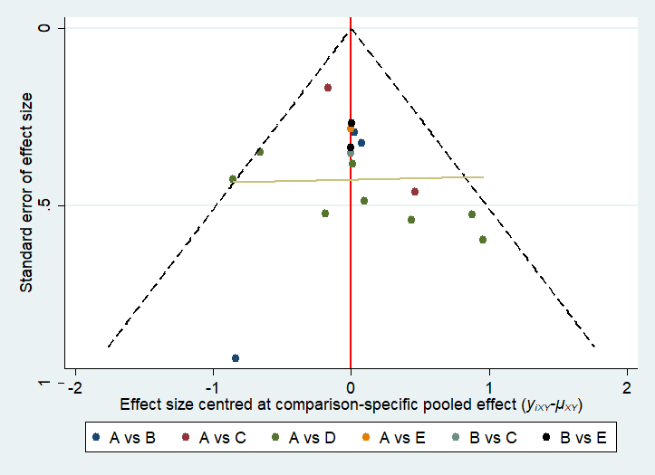


Supplemental 3.12 Comparison-adjusted funnel plot for live birth rate.

A: Placebo/blank, B: Cabergoline, C: Albumin, D: Metformin, E: Calcium.
